# Evaluation of cell adhesion and osteoconductivity in bone substitutes modified by polydopamine

**DOI:** 10.3389/fbioe.2022.1057699

**Published:** 2023-01-13

**Authors:** Ali Mahnavi, Mina Shahriari-Khalaji, Bahareh Hosseinpour, Mostafa Ahangarian, Amir Aidun, Simona Bungau, Syed Shams ul Hassan

**Affiliations:** ^1^ Department of Biomaterials, Faculty of Interdisciplinary Science and Technology, Tarbiat Modares University, Tehran, Iran; ^2^ State Key Laboratory for Modification of Chemical Fibers and Polymer Materials, College of Materials Science and Engineering, Donghua University, Shanghai, China; ^3^ Department of Chemistry, Carleton University, Ottawa, ON, Canada; ^4^ National Cell Bank of Iran, Pasteur Institute of Iran, Tehran, Iran; ^5^ Tissues and Biomaterials Research Group (TBRG), Universal Scientific Education and Research Network (USERN), Tehran, Iran; ^6^ Department of Pharmacy, Faculty of Medicine and Pharmacy, University of Oradea, Oradea, Romania; ^7^ Shanghai Key Laboratory for Molecular Engineering of Chiral Drugs, School of Pharmacy, Shanghai Jiao Tong University, Shanghai, China; ^8^ Department of Natural Product Chemistry, School of Pharmacy, Shanghai Jiao Tong University, Shanghai, China

**Keywords:** biomaterials, scaffold, tissue engineering, bone, polydopamine, surface modification, cell adhesion, osteoconductivity

## Abstract

Bones damaged due to disease or accidents can be repaired in different ways. Tissue engineering has helped with scaffolds made of different biomaterials and various methods. Although all kinds of biomaterials can be useful, sometimes their weakness in cellular activity or osteoconductivity prevents their optimal use in the fabrication of bone scaffolds. To solve this problem, we need additional processes, such as surface modification. One of the common methods is coating with polydopamine. Polydopamine can not only cover the weakness of the scaffolds in terms of cellular properties, but it can also create or increase osteoconductivity properties. Polydopamine creates a hydrophilic layer on the surface of scaffolds due to a large number of functional groups such as amino and hydroxyl groups. This layer allows bone cells to anchor and adheres well to the surfaces. In addition, it creates a biocompatible environment for proliferation and differentiation. Besides, the polydopamine coating makes the surfaces chemically active by catechol and amine group, and as a result of their presence, osteoconductivity increases. In this mini-review, we investigated the characteristics, structure, and properties of polydopamine as a modifier of bone substitutes. Finally, we evaluated the cell adhesion and osteoconductivity of different polydopamine-modified bone scaffolds.

## 1 Introduction

Bone defects and bone deficiency caused by removing tumors or infections are one of the main problems in orthopedics, oral and maxillofacial, and Implantology surgeries. Bone grafting, therefore, is used as an efficient method for treating and regenerating to stabilize fixtures and implants and complete restoration (Salyer and Taylor, 2009). Bone grafting cannot be used just for treating defected bone but also as a scaffold to improve osteoconductivity and cell adhesion in the purpose area (Lee et al., 2021). Nowadays, there are manifold procedures and new technologies for manufacturing and modification of foreign bone substitutes due to achieve the best results, for example, deproteinization and delipidation of allogenic, and also xenogenic bones (Gardin et al., 2015). A cutting-edge bioengineering method is modifying surfaces of bone substitutes that can improve cell adhesion affinity rate, biodegradability, and biocompatibility (Ghorbani et al., 2019a; Kheilnezhad et al., 2020). Modified bio substitutes such as orthopedic implants that have significantly increased antibacterial and osteoconductive properties by surface modification can play a unique role in promoting bone tissue engineering in the compare with the other materials (Li et al., 2015; Hosseini et al., 2019).

Biopolymers are one of the most potent substances to make a chemical layer to guide cell proliferation, adhesion, and differentiation (Aidun et al., 2019a; 2019b; Ghorbani et al., 2019b). One of the most reliable and functional biopolymers for improving surface properties is PDA (Polydopamine), or polymerized dopamine which has been used as an assistant for surface modification by making a super hydrophilic substrate on a biomaterial interface. This coating is created by an oxidation reaction of dopamine (Lynge et al., 2011). PDA also is comfortably available, and by exposing dopamine to the air in a buffer solution manufactured, and can be linked to metals, oxides, ceramics (Lee et al., 2007), biopolymers (Ghorbani et al., 2019a), carbon nanotubes (Demirci et al., 2021), and magnetic nanoparticles for drug delivery (Si and Yang, 2011; Mrówczyński et al., 2013). Up to now, many structures of PDA have been introduced, but it is fascinating that precise structure has not been ultimately discovered yet (Liebscher et al., 2013).

This article focuses on PDA-modified scaffolds characterization and application for bone tissue regeneration. Firstly, we discussed the properties of different bone substitutes. Furthermore, we report researches carried out on types of microstructures of PDA used for surface modification.

## 2 Introduction of bone substitutes materials

The bone augmentation process is based on several factors. Osteoblasts, osteoclasts, and their precursors are the provenance of this action (Lischer, 2019). Most bone substitute materials act as a scaffold structure for the osteoconductive ingrowth of osteoblasts and mesenchymal progenitors (SafaeiFiroozabady et al., 2019; Siswanto et al., 2020; Hu J. et al., 2021). Various materials are used in bone tissue engineering as substitutes and scaffolds, either alone such as biopolymers, metals, and ceramics, or in composite form. These fabricated scaffolds sometimes need modification in various ways to improve some properties such as mechanical properties, cellular behavior, or osteoconductivity (Zhang et al., 2017; Ghorbani et al., 2019a; Nosrati et al., 2021).

### 2.1 Metals

Research showed that metallic materials and their alloys have been accepted as an implant in dentistry and spinal surgery for a few decades (Glenske et al., 2018). Aluminum, in turn, is associated with toxic adverse effects, including anemia, encephalopathy, and osteoporosis (Aaseth et al., 2012; Mozafari et al., 2020). Despite these drawbacks, the composition of aluminum (Al) with Titanium (Ti6Al4V) is widely used as a dental and orthopedic implant. While PDA substrate is formed on Ti6Al4V, a strong interaction between the coating layer and Ti leads to enhanced mechanical properties and corrosive resistance (Hu Y. et al., 2021). Moreover, modification with PDA can increase osteoconductivity and bioactivity properties in metals and their alloys, and also make a more straightforward condition for the differentiation of human mesenchymal stem cells (hMSCs) to endothelial cells (Yu et al., 2014). By immersing the titanium-based scaffolds in prepared dopamine solution for 24 h in the darkroom at room temperature, usually a thin layer of PDA forms on the surface of the scaffolds. The use of oxidation agents can reduce the coating time with PDA. Also, the molar ratio between dopamine and the oxidation agent can bring different characteristics for the scaffold, for example, antibacterial properties or different levels of cytotoxicity. Among oxidizing agents, potassium permanganate (KMnO_4)_ showed possible antipathogenic effects, and in the molar ratio .45:.88 (KMnO_4_:dopamine) the sample manifested suitable antimicrobial properties, while in the molar ratio .1:1.5 (KMnO_4_:dopamine) the samples led to more nitric oxide release and being some cytotoxic (Marinas et al., 2021). Li et al. (2019) showed in their study that PDA coating on the 3D printing porous Ti6Al4V scaffolds significantly improves MC3T3-E1 cell attachment after 4 h. They also investigate the osteogenesis by *in-vivo* evaluation on the adult rabbits within 3–5 kg and the result showed that the PDA-coated group had significantly (*p* <.01) more newly formed bone tissue and this is evidence of the osteoconductivity of the PDA.

### 2.2 Biopolymers

Poly (l-lactide-co-glycolide) (PLGA) (Zhang et al., 2017), polycaprolactone (PCL) (Aidun et al., 2019a), poly (L-lactide) (PLLA) (Liu et al., 2019), Poly (methylmethacrylate) (PMMA) (Soleymani Eil Bakhtiari et al., 2021), collagen (Aidun et al., 2019a), polyurethane (Aidun et al., 2019b), and polysaccharides (Aidun et al., 2019c) are biomaterials with various applications. These biomaterials are widely used as substitutes in bone tissue engineering. Although they have suitable biodegrading and biocompatibility, they have no appropriate osteoconductivity (Rocha et al., 2022). Most of them can be used as just bioactive molecules or growth factor carriers (Eap et al., 2015). Research indicated that the properties of their surface can be modified by PDA and the osteoblast growth and after modification by PDA has significantly increased. Ko et al. (2013) in their study showed that PDA can play an important role to immobilize osteoinductive molecules such as growth factors or peptides. They could immobilize bone morphogenetic protein-2 (BMP-2) on the PLGA scaffold by PDA (Ko et al., 2013).

### 2.3 Ceramics

Ceramics have been used since the 1980s in orthopedics and dentistry and are also made from calcium phosphate (Bohner, 2000). Calcium phosphate ceramics (CPCs) are a kind of bioactive material that has been used for bone tissue engineering. They have a surface that supports cell adhesion, leading to increased osteoconductivity. However, most CPCs have an osteoconductive property but possess different cell growth abilities (Samavedi et al., 2013). Fan et al. (2022) in their study evaluated the effect of the PDA coating and chitosan (CS) coating on the 3D-printed biphasic calcium phosphate (BCP) scaffold. They assessed the cell behavior of the scaffold and showed that MC3T3-E1 cells after 2 days were attached to the BCP/PDA with longer pseudopodia compared with BCP. The Relative gene expression of Runx-2 and COL-1 were significantly (*p* <.05) higher on BCP/PDA than BCP which indicated that PDA could promote the osteogenic differentiation of MC3T3-E1. The osteoconductivity and mineralization of the scaffold observed and the morphology of the surface mineralization of the scaffolds after 2 weeks showed that the volume of apatite formed in BCP/PDA was more than in BCP.

## 3 Microstructure and sources of PDA

### 3.1 Source of PDA

PDA is a polymer inspired by adhesive proteins left in mussel plaques, Because, they are able to adhere to many substances ranging from wood to stones (Ding et al., 2016). Mussel’s plaques are rich in a catechol named 3,4-dihydroxy-L-phenylalanine (DOPA) and lysine amino acids. Therefore, the existence of both amino acids and catechol is the cause of the strong adhesive ability in mussels (Lee et al., 2007). PDA includes both two mentioned groups and Lee et al. (2007) have discovered a method to form PDA coating on surfaces through the simple coating of objects in an aqueous solution of dopamine. Forming of PDA is indicated below.

### 3.2 Structure of PDA

A large number of structures of PDA are introduced, but to better understand precise PDA structure, the mechanism involved in PDA formulation should be explained. At first, the oxidation of dopamine into dopamine-o-quinone (DQ) made in alkaline PH conditions is noticeable (Velez-Pardo et al., 1995; Lee et al., 2007; Ho and Ding, 2014; Wu et al., 2021). Then, because of the deprotonation of the primary amine, DQ by an irreversible cycle through 1,4 Michael addition converts into leukodopaminechrome (LDC) (Li et al., 2006; Lee et al., 2007). Finally, LDC oxidizes to dopaminechrome (DC) then it isomerizes to 5,6-dihydroxyindole (DHI) before oxidation of DHI to 5,6-indolequinone (IDQ), which is the monomers of PDA (Bisaglia et al., 2007).

The carbon at 2, 3, 4, and 7 conditions in both DHI and IDQ can undergo branching reactions to form different isomers of oligomers and further self-assemble to form PDA. For the oxidation process, solvable oxygen is a required part of these reactions (Ho and Ding, 2014). Although there is a well-defined monomeric progenitor, the precise mechanism of PDA formation is still under discussion, and some researchers believe that the PDA structure might be similar to eumelanin (Watt et al., 2009; Yu et al., 2010; Chen et al., 2013).

## 4 Super-hydrophilic layer in composites, biopolymers, and ceramics scaffolds with PDA

Dopamine polymerization results in PDA fabrication which can be coated in different substrates including noble metals, oxides, ceramics, biopolymers, and semiconductors. The PDA layer which has a tunable thickness (10–100 nm) could provide valuable properties for coated surfaces including high hydrophilicity, long-term corrosion resistance, and moderate physicochemical properties (Huang et al., 2016). The dopamine concentration, the temperature of the dopamine solution, the speed of the stirrer, oxidative factors such as potassium permanganate, and the scaffold immersion time in the dopamine solution could affect the thickness of the PDA layer and the time of the PDA coating. Based on the literature, The polydopamine layer will be larger at higher concentrations of dopamine, higher solution temperature, and higher stirrer speed (Terrill, 2015; Oymaci et al., 2020).

Wettability of the surface could affect the surface protein adsorption and also cell attachment. Cells tend to attach to the hydrophilic surface and by increasing the contact angle from 0° to 106° the rate of cell attachment decreases and the most rate of fibroblast cell attachment on the surface is between 60°–80° based on the literature. But on the super-hydrophilic surface with a contact angle under 5° is unsuitable for proteins and cells to bind and attach. Super-hydrophilic surfaces may affect the structure, type, and binding strength of the proteins such as albumin, transferrin, and fibronectin from the cell culture medium and this could have a negative effect on the cell attachment (Arora, 2013). Besides, a super-hydrophobic surface with a contact angle of more than 150° could have the same manifestation, and the proteins such as laminin, collagen, and fibronectin in extra cellular matrix adsorbed denatured and this also prohibits cells from adhering to the surfaces (Cai et al., 2020). It has been reported attachment of NIH3T3 fibroblast cells was enhanced following PDA coating (Sun et al., 2011). In another study, Sun et al. (2012) revealed that coating the PDA layer on bio-substitutes could impressively improve the immobilization of rabbit chondrocytes. Table 1 presents more results of other studies.

**TABLE 1:** Hydrophilic layer on scaffolds with PDA.

Composite	Hydrophilic Layer	Contact angle bofore PDA modification	Contact angle after PDA modification	Cell type	Cell behavior	Results	Ref
Copoly(phthalazinone biphenyl ether sulfone) (PPBES)	PDA-assisted hydroxyapatite formation (pHAF)	73.2° ± 2.7°	56.7° ± 0.7°	NIH-3T3	After coating, cells spread better and showed more flattened morphology	The coating enhances cytocompatibility in vitro. Cell attachment significantly increased (*p* < 0.05) (Figure 1A)	Liu et al. (2018)
Polydimethylsiloxane (PDMS)	PDA	109°	86.8°	Bone marrow mesenchymal stem cells (BMSCs) and chondrocytes	BMSCs showed suitable differentiation in the specific area of the surfaces, at the edges of micro-nano patterns	PDMS/PDA with a better biocompatibility than PDMS for cartilage and bone tissue engineering	Cao et al. (2021)
PLLA nanofibers	PDA	122.5°±7.3°	32.7° ± 3.9°	hMSCs	In the PDA-coated scaffold, cell attachment and proliferation improved compared with the simple scaffold. Cell attachment and proliferation were better than other groups.	The results demonstrated the PLLA-PDA-osteogenic growth peptide (OGP) scaffold has a promising capacity for the repair of bone with the critical defect.	Liu et al. (2019)
Hydroxyapatite/polyamide 66 (HA/P66)	PDA	70.22°±3.67°	42.98°±1.99°	C3H10T1/2	On ccK-8 assay for cell attachment, the optical density (OD) of PDA-HA/P66 was significantly more than HA/P66 (*p* < 0.05). Based on the SEM images, the cells spread better on the PDA-HA/P66 compared with HA/P66. The expression of COL-1, RUNX2, and OCN of PDA-HA/P66 was higher (*p* < 0.05) than that of HA/P66	The alkaline phosphatase (ALP) activity of PDA-HA/P66 was higher (*p* < 0.05) than HA/P66. Based on the in-vivo and in-vitro evaluation, the coated scaffolds improved the osteogenic-inducing ability of the scaffolds and could be a good way for surface modification for bone substitution	Xu et al. (2018)
Hydroxyapatite-collagen calcium silicate (HCCS)	PDA	51.43°± 1.79°	35.90°±3.51°	Rat mesenchymal stem cells (rMSCs)	The HCCS-PDA samples showed a minimum range in Live/Dead cytotoxic assay and demonstrated higher cell attachment and proliferation than the HCCS	The results indicate that the HCCS-PDA biomaterial could apply as an osteoconductive scaffold material, with potential for more expansion into therapeutic bone tissue engineering strategies and clinical applications	Lee et al. (2017)
					samples in vitro		
Polyetheretherketone (PEEK)	PDA	91.00°±1.60°	78.73°±3.15°	Rat BMSCs	Results of ccK-8 for Cell proliferation indicated BMSCs exhibited significantly higher vitality in all PDA-coated groups (*p < 0.05)	PDA-coated scaffolds become more hydrophilic which helps cellular responses and also functional protein adsorption. PDA may be used as a functional bioactive coating in bone regeneration and orthopedic applications	Wang et al. (2019)
					Results of the early marker of osteoblast differentiation (ALP) in PEEK-PDA sample showed more higher ALP activity compared with the uncoated samples (*p < 0.05)		
Biphasic Calcium Phosphate (BCP)	PDA	40.6° ± 1.8°	10.5 ± 1.4°	BMSCs	The viability of cells was identified by live/dead fluorescent staining and it showed high viability, with almost no dead cells on PDA-modified scaffolds	Based on the results, digital light processing (DLP)-printed BCP bioceramic scaffolds coated with PDA/BMP-2 could be a promising strategy to fabricate bone substitute scaffolds (Figure 1D)	Yang et al. (2022)
					Cells on the BMP-2/PDA-BCP scaffold were significantly more and also exhibited a higher proliferation rate compared with the simple scaffold on Day 7		
Silk Fibroin (SF)/Curcumin(CM)	PDA	81.02°± 0.35°	60.17°± 0.17°	osteoblast MC3T3-E1 and osteosarcoma MG-63	SF/CM-PDA inhibited the growth of the MG-63 and also promotes the proliferation of MC3T3-E1 cells in vitro	Based on the result	Meng et al. (2021)
						SF/CM-PDA biomaterials scaffolds have the superb potential for the treatment of osteosarcoma and tumor-related bone defect repair	
micro-arc oxidation coating doped with HA particles (MAO-HA)	PDA	100.5°	80.1°	Osteoblast MC3T3-E1	After 24 h, cells spread over the surface as expected with prominent lamellipodia extensions and also Filopodia	According to the result, PDA coating promoted the bioactivity and biological properties of Mg alloys and it could be a potential option for surface modification for bone tissue engineering applications	Feng et al. (2017)

According to the literature, PDA has a positive effect on increasing the hydrophilicity of the surface of the scaffold. Rezaei et al. (2021) showed in their study that PDA coating could significantly improve the contact angle and absorption capacity (*p* <.005) of the PLGA-Gelatin (PG) lamellar composite scaffold. The contact angle of the PG scaffold and PDA-coated PG were 61.77° ± .65° and 32.05° ± .86°, respectively. The results of this study indicated that the PDA coating and lamellar structure have a synergistic effect on physicochemical properties such as hydrophilicity.

Recently, PDA is also used specifically in the field of 3D printers for bone tissue engineering. Feng et al. (2021) investigated the effect of the PDA coating in a 3D-printed scaffold. They fabricated the 3D scaffolds with the PDA functionalized PCL powder *via* a selective laser sintering printer. The results indicated that the scaffolds have abundant catechol groups on the surfaces and also show perfect hydrophilicity because of the specific functional group such as amino and hydroxyl groups.

## 5 Assessment of osteoconductivity and cell adhesion rate in different types of PDA-modified bone substitutes

Bone grafts, such as autografts, allografts, and xenografts, are frequently used in clinical treatment, although their performance needs to be improved. The optimum scaffold for bio-replacement materials in bone defects intends to promote cell proliferation, osteo-differentiation, and mineralization, which can be achieved by PDA-assisted surface modification (Huang et al., 2016).

A unique, powerful charge transfer interaction could be developed between Ti and the coated surface by applying the PDA layer to the bio-substitutes surface, giving excellent corrosion resistance, higher mechanical properties, and great superficial energy. Furthermore, the PDA-assisted surface modification of Ti and Ti alloys can provide bioinert metallic bone bio-substitutes with osteoinductivity and osteoconductivity, and improve their bioactivities in terms of cell viability, proliferation, and differentiation based on the Yu et al. (2014) study. The result of their study indicated that the PDA and collagen coating have a positive effect on cellular behavior such as cell attachment, proliferation, and also differentiation of the preosteoblasts. The number of cells was significantly (*p* <.05) higher in PDA-coated groups compared with the control group. The same result was found on the treated surfaces in the ALP activity and the PDA-coated scaffold showed significantly (*p* <.05) more ALP activity compared with the pristine Ti (Yu et al., 2014).

PDA is one of the mineral inducers that helps to produce mineralized surfaces. With the help of the PDA-coated layer, the hydroxyapatite-coated bio-substitute surface (such as titanium alloy, Ti6Al4V) can immobilize some active biomolecules like BMP-2. These immobilized biomolecules can then be evenly distributed on the surface and show a sustainable release profile when used in a natural environment for bone repair. Cai et al. (2014) showed in their research the role of the PDA in the immobilization of BMP-2 on the surface. They showed the ALP activity was higher significantly in three groups of Ti-PDOP (PDA-grafted Ti6Al4V), Ti-H (Ti-PDOP immersed in the 1.5SBF for 5 days), and Ti-BMP2 (BMP2 peptide absorbed Ti-HA) than in the pristine substrate (*p* <.05). Also in this research, the number of the adherent cell on the surface of the Ti-BMP2 group was significantly higher than Ti (*p* <.05, X ± SD, *n* = 3).

It has been reported that PDA-coated layers can control cellular behaviors, such as enhanced osteoblast cell proliferation and calcium deposition. This impact might be further amplified when growth factors are combined. It has also been widely reported that the PDA coating layer on bio-substitutes improved the immobilization of rabbit chondrocytes; another report stated that PDA coating layers could impressively enhance the attachment of NIH3T3 fibroblast cells (Sun et al., 2012). Moreover, PDA can be employed as a controlled surface modifier to strengthen the scaffold’s cell affinity or biomaterial’s surface selective antifouling (Li et al., 2018). VEGF may also be efficiently immobilized on the Ti-based bio-substitute by the PDA coating layer without impairing VEGF activity for bone implants (Poh et al., 2010). Such layer-by-layer deposited composite bone bio-substitute comprising VEGF, PDA layer, and Ti material significantly promotes endothelial cell adhesion and proliferation. Poh et al. (2010) showed in their study the human dermal microvascular endothelial cells (HDMEC) cell proliferation on the Ti-PDOP-VEGF (VEGFcoated, PDA-grafted Ti) was significantly higher than the cells on the simple Ti (*p* <.05). Also, the number of cells on the Ti-PDOP-VEGF was about 20% more than the number of attached cells on the pristine Ti which is statistically significant (*p* <.05). According to this study, HDMEC was used due to the major role of revascularization around the implant. The goal of PDA-assisted surface modification on various metallic surfaces is to introduce novel concepts and processing strategies to improve revascularization during bone regeneration considering various functions obtained by immobilizing different biomolecules.

**FIGURE 1:**
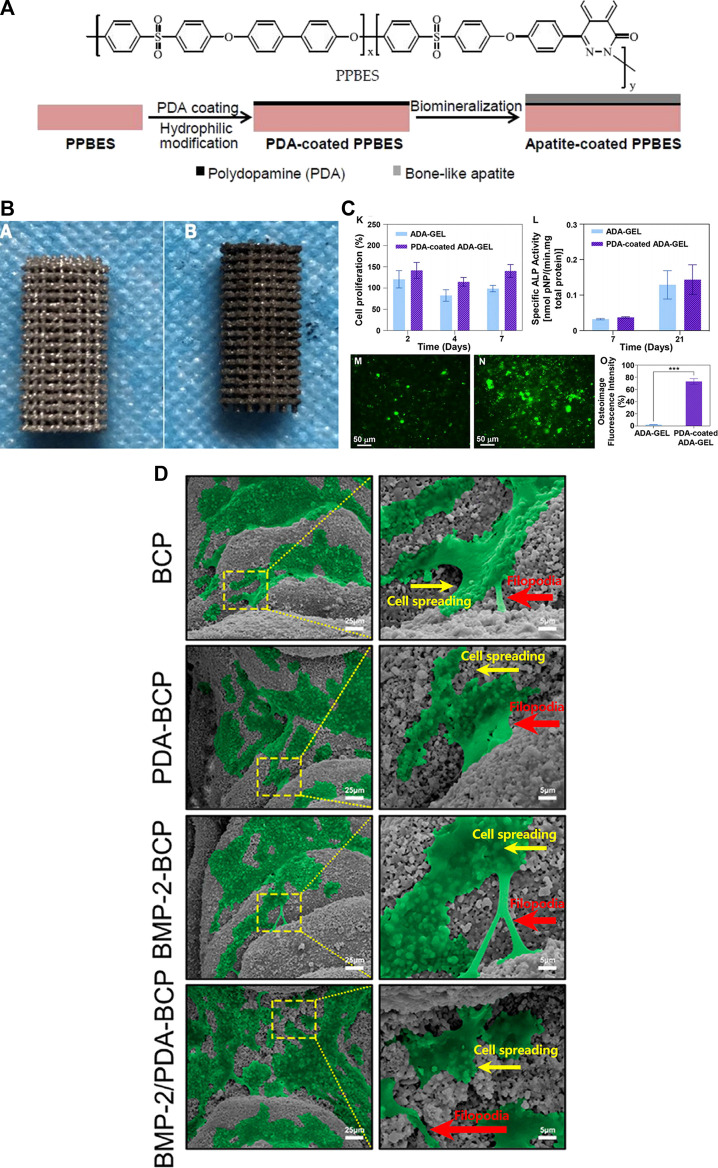
**(A)** The structure of Copoly (phthalazinone biphenyl ether sulfone) (PPBES) and the dopamine polymerization on the PPBES, and finally apatite biomineralization on the surface. [reproduced content is open access- Attribution 4.0 International (CC BY 4.0)] (Liu et al., 2018). **(B)** The image of the 3D-printed porous titanium (3D PPT) (a), and the PDA coating on 3D-printed porous titanium (PDA-3D PPT) (b). (reproduced content is open access- Attribution 4.0 International (CC BY 4.0)) (Zhong et al., 2020). **(C)** The proliferation of the MG-63 osteoblast-like cells on the ADA-GEL, and PDA-coated ADA-GEL scaffolds at the 2, 4, 7 days. Although the proliferation of coated scaffolds increased, this increase was not significant (K), The ALP activity of the ADA-GEL, and PDA-coated ADA-GEL scaffolds at the 7, and 21 days. Although the ALP activity of coated scaffolds increased, this increase was not significant (L). (reproduced content is open access- Attribution 4.0 International (CC BY 4.0)) (Ghorbani et al., 2022). **(D)** Osteoimage mineralization of the uncoated ADA-GEL scaffold (M) and PDA-coated ADA-GEL scaffold (N). The result of the quantitive osteoimage mineralization. The result was significant (***p <.001) (reproduced content is open access- Attribution 4.0 International (CC BY 4.0)) (Yang et al., 2022).

Synthetic and bioderived biopolymers, such as collagen (Aidun et al., 2019a), starch (Aidun et al., 2019c), and PCL (SafaeiFiroozabady et al., 2019) are another important class of materials used to create bone bio-substitute. Although most polymeric biomaterials have adequate mechanical properties, biodegradability, biocompatibility, and erosion resistance, they do not provide good osteointegration (Rocha et al., 2022). PDA-assisted surface modification can significantly improve the surface properties such as the osteogenesis activity of these biopolymers (Zhou et al., 2017). The ability of osteoblasts to respond to polymeric materials can be markedly enhanced in the presence of immobilized BMP, and the material mineralization can be accelerated in the presence of immobilized growth factors (Pan et al., 2014). Bone filler materials are used in a variety of bone tissue engineering applications, including plastic surgery and bone defects. The cytoaffinity of poly (dimethylsiloxane)-based bone cement can be improved by adding a PDA coating layer to poly (dimethylsiloxane), allowing NIH3T3 fibroblasts’ adherence to the material substrate more easily and thus promoting cell growth (Jun et al., 2014). In a study, Zhong et al. (2020) evaluated the effect of the PDA coating on the 3D-printed titanium for repairing a femoral defect in rabbits. They show on the micro-CT after 12 weeks, the bone volume values of the PDA-coated 3D printed porous titanium (PDA-3D PPT) are significantly higher than the uncoated scaffold and also blank group (*p* < .05). The uncoated and PDA-coated of the 3D-printed scaffold were illustrated in Figure 1B.

Besides that, the developed PDA coating layer may provide tunable regulating features by mediating numerous parameters, such as multiple cell lines, PDA preparation, and cultivation of cells under various settings. For example, altering the dopamine (PDA precursor) incubation temperature may augment the ratio of quinone to phenolic hydroxyl groups, which will help control the proliferation of smooth muscle cells linked to the bio-substitutes (Luo et al., 2013). Ghorbani et al. (2022) indicated the role of quinone in their study. They showed that the PDA-coated alginate dialdehyde-gelatin (ADA-GEL) is functionalized by various groups which makes the surface of the 3D printed scaffold suitable for biological properties such as cell attachment and proliferation. The results of osteoimage mineralization in the PDA-coated group were significantly higher than the uncoated scaffold after 21 days of cell culture (***p <.001) (Figure 1B). Osteoimage fluorescence intensity of ADA-Gel and PDA-coated ADA-Gel showed 2.5% and 75%, respectively. Although the MG-63 proliferation and also ALP activity of the PDA-coated groups are higher than the uncoated group, these increases were not statistically significant. Due to the fact that the ALP activity plays a crucial role in the preosteoblastic stage of bone cell differentiation, this could be an indicator of osteoblastic differentiation and osteoconductivity of the scaffold (Ghorbani et al., 2020).

Growth factors, which are sensitive biomolecules easily rendered inactive by environmental changes, can be immobilized on PDA-coated bio-substitutes with little or no effect on their functions. The generated PDA coating layer allows growth factors like BMP (Zhang et al., 2017), and Arg-GlyAsp peptide (RGD) (Jeong et al., 2011) to bind with the surface of bio-substitutes. According to studies (Jeong et al., 2011; Cho et al., 2014; Ghorbani et al., 2019a), only the PDA coating layer forming conditions and the inherent characteristics of biomolecules (incubation time, concentration, molecule weight, and isoelectric point) had an impact on the activity of these biomolecules. PDA-assisted immobilization has the potential to significantly improve biomolecules’ ability to anchor when compared to conventional immobilization methods (Vanderleyden et al., 2014). The specific functional groups such as amino and hydroxyl could enhance the proliferation of the MG-63 cells and also cell attachment based on the Feng et al. (2021) study. They pointed out that the presence of these functional groups is due to the presence of catechol and amine groups in primary PDA. Besides, the abundance of catecholamine moieties in PDA has a special role in osteoconductivity. The mechanisms of osteogenesis virtually are related to the catecholamine’s location on the interfaces and also the Ca^2+^ ion binder which forms and expedites the following hydroxyapatite crystals on the surface of the PDA-coated materials (Ghalandari et al., 2021). PDA functionalize chemically the surface and also plays an important role in creating new hydroxyapatite nuclei on the surfaces of the scaffold (Larrañaga et al., 2015).

## 6 Conclusion and discussion

According to the superb physicochemical and biological properties of PDA, it could use as a scaffold surface modifier in bone tissue engineering. PDA is virtually a suitable material to coat the surface of the scaffold. Based on numerous studies, it enhances osteoconductivity and also cell adhesion and could be a game changer, especially in bone tissue engineering. Since the properties of PDA are excellent for bone tissue engineering, most of the performed studies are at the *in-vitro* and *in-vivo* stages. The main limitation is that there is a lack of clinical studies on this subject until now. With the increase in the number of pre-clinical studies on this functional material in the field of bone tissue engineering, more information will be obtained, which will be of great help in conducting clinical studies. A large number of studies show that PDA has the potential to be used in coating different types of dental implants. It also has a high potential in the treating and healing of bone fractures with the help of various scaffolds such as PDA-coated 3D scaffolds. Looking at the limitations and potentials for the use of PDA, there is a bright future for it.
